# Lower Uterine Segment Compression Sutures for Hemorrhage During Cesarean Delivery in Women With Placenta Previa: A Systematic Review of Hemostatic Success and Uterine Preservation

**DOI:** 10.7759/cureus.117789

**Published:** 2026-10-11

**Authors:** Jawariya Marfani

**Affiliations:** 1 Obstetrics and Gynecology, Edinburgh Royal Infirmary, Edinburgh, GBR

**Keywords:** cesarean delivery, hemostasis, lower uterine segment compression suture, placenta previa, postpartum hemorrhage, uterine preservation

## Abstract

Bleeding from the lower uterine segment is difficult to control during cesarean delivery for placenta previa, and conventional compression sutures were designed mainly for uterine atony. We systematically reviewed comparative studies of lower uterine segment compression sutures, focusing on hemostatic success and uterine preservation.

Following Preferred Reporting Items for Systematic Reviews and Meta-Analyses (PRISMA) 2020, PubMed, Scopus, Web of Science, and Embase were searched through 12 September 2026. Original comparative studies from the last five years that included women with placenta previa undergoing cesarean delivery were eligible. Risk of bias was assessed with the Risk of Bias in Non-randomized Studies - of Interventions (ROBINS-I). Because techniques and comparators differed, results were synthesized narratively without pooling.

Of 183 records identified, four retrospective comparative cohorts (91-346 women) were included. In the two studies reporting mean blood loss, suture groups lost less blood than comparators (2152.6 versus 2960.5 mL; 792.5 versus 1220.8 mL). Operative times were shorter, and fewer additional procedures were needed. Hysterectomy was infrequent: none occurred with double-row transfixion versus two with aortic balloon plus sutures. Two studies had a low overall risk of bias and two a serious risk.

Lower uterine segment sutures appear to be a useful, low-cost hemostatic option in placenta previa, but the evidence is limited to four retrospective cohorts. Prospective comparative studies with standardized outcomes are needed.

## Introduction and background

Placenta previa, in which the placenta implants over or close to the internal cervical os, is a leading obstetric cause of antepartum and intrapartum hemorrhage, and its occurrence rises with prior cesarean delivery and advancing maternal age [1]. When previa coexists with placenta accreta spectrum (PAS), the abnormal trophoblastic invasion of the lower uterine segment markedly raises the risks of massive hemorrhage, transfusion, peripartum hysterectomy, and maternal morbidity [2]. In previa without PAS, bleeding arises mainly from the placental bed after normal placental separation, whereas in previa with PAS, the placenta is adherent or invasive, separation is incomplete, and hemorrhage is heavier and harder to control [2]. For both groups, cesarean delivery is often the moment at which bleeding becomes life-threatening, particularly after the placenta is separated and the placental bed is exposed.

The anatomy of the lower uterine segment explains why conventional measures frequently fail. This region contains less smooth muscle than the uterine body, contracts poorly after delivery, and cannot reliably compress the vessels of the placental bed, so uterotonic drugs and uterine massage may be ineffective. Uterine compression sutures were introduced to preserve the uterus when medical measures fail, and the B-Lynch and Hayman techniques are the best known [3]. Their role in postpartum hemorrhage has been summarized in a comprehensive overview, but they were designed largely for the atony of the uterine body and may not reach the bleeding surface in the lower segment [4]. Surgeons have therefore developed segment-specific variants, including transverse, parallel vertical, circular, and transfixion sutures, some combined with intrauterine tamponade [5]. These sutures are placed across or through the lower segment itself, apposing its anterior and posterior walls and compressing the placental bed directly, which sutures placed on the uterine body cannot achieve.

Despite their widespread use, the evidence base for segment-specific sutures is fragmented. Most reports are single-center technical descriptions or small case series, outcomes are defined inconsistently, and comparators range from conventional oversewing to balloon tamponade or vascular occlusion. Hysterectomy, the outcome that matters most to women who wish to preserve fertility, is not always reported. A synthesis restricted to studies with a comparison group could clarify whether these sutures improve hemostasis and uterine preservation. We therefore systematically reviewed comparative studies published in the last five years on lower uterine segment compression sutures during cesarean delivery for placenta previa, with hemostatic success and uterine preservation as the principal outcomes.

## Review

Methods

Study Design

This systematic review was conducted and reported in accordance with the Preferred Reporting Items for Systematic Reviews and Meta-Analyses (PRISMA) 2020 statement [6].

Eligibility Criteria

Eligibility was defined using the population, intervention, comparator, outcomes, and study design (PICOS) framework (Table 1). Only original, peer-reviewed comparative studies published in the last five years (September 2021 to September 2026) were included.

**Table 1:** PICOS eligibility criteria PAS, placenta accreta spectrum; PICOS, population, intervention, comparator, outcomes, and study design

Element	Inclusion	Exclusion
Population	Women with placenta previa (with or without PAS) undergoing cesarean delivery	Mixed populations without separate previa data
Intervention	Lower uterine segment compression or transfixion suture (with or without local tamponade)	Techniques based on vascular occlusion or embolization
Comparator	Any other hemostatic approach	No comparator
Outcomes	Hemostasis or blood loss; hysterectomy or uterine preservation	No relevant clinical outcomes
Study design	Original comparative studies	Reviews, editorials, abstracts, letters, protocols, preprints, case reports, and single-arm series

Information Sources and Search Strategy

PubMed, Scopus, Web of Science, and Embase were searched for records published from September 2021 to 12 September 2026, the date of the last search. Search terms combined placenta previa, the lower uterine segment, compression or transfixion sutures, and cesarean delivery, using controlled vocabulary (MeSH and Emtree) and free-text words adapted to each database. The complete database-specific strategies are given in the Appendices.

Selection Process

Duplicate records were removed. Records were screened by title for relevance, and the full texts of retained reports were assessed against the eligibility criteria. Reasons for exclusion at the full-text stage were recorded and are shown in the PRISMA flow diagram. Each excluded report was assigned to one predefined reason (not related to the lower uterine segment; review, editorial, or abstract without full data; no relevant clinical outcome). Screening, full-text assessment, data extraction, and risk-of-bias assessment were performed by a single reviewer.

Data Collection Process and Data Items

Data were extracted from each included study using a standardized form. The primary outcomes were hemostatic success or blood loss and hysterectomy or uterine preservation. Secondary outcomes were operative time, transfusion, hospital stay, and complications. We also extracted first author, year, country, study design, population, sample size, intervention, and comparator.

Study Risk-of-Bias Assessment

Risk of bias was assessed with the Risk of Bias in Non-randomized Studies - of Interventions (ROBINS-I) tool across seven domains: confounding, the selection of participants, the classification of interventions, deviations from intended interventions, missing data, the measurement of outcomes, and the selection of the reported result [7]. Each domain and the overall risk were rated as low, moderate, or serious.

Effect Measures and Synthesis Methods

Continuous outcomes such as blood loss and operative time were extracted as reported means with standard deviations and categorical outcomes such as hysterectomy as counts. Where means, standard deviations, and group sizes were reported, the mean difference in blood loss and its 95% confidence interval (CI) were calculated with the Welch method, together with the percentage reduction; P values are those reported in the original studies. Quantitative pooling was considered inappropriate because the studies tested different suture techniques against different comparators (figure-of-eight sutures, balloon tamponade, aortic balloon with sutures, and uterine packing), enrolled populations with different PAS prevalence, and defined blood loss and hemostatic success differently. Only two studies reported blood loss as means with standard deviations and against different comparators. Each study was therefore summarized separately as a within-study comparison and tabulated by design and comparator, and no summary estimate across studies was calculated. No meta-analysis was performed.

Reporting Bias Assessment

Selective outcome reporting was evaluated within the ROBINS-I framework. A formal assessment of publication bias, such as a funnel plot, was not feasible with so few studies.

Results

Study Selection

The search identified 183 records (PubMed, 42; Scopus, 37; Web of Science, 32; Embase, 72). After the removal of 96 duplicates, 87 records were screened by title, and 51 were excluded. All 36 reports sought for retrieval were obtained and assessed for eligibility. Thirty-two were excluded: 13 were not related to the lower uterine segment; 17 were reviews, editorials, or abstracts without full data; and two lacked relevant clinical outcomes. Four studies were included (Figure 1) [8-11].

**Figure 1:**
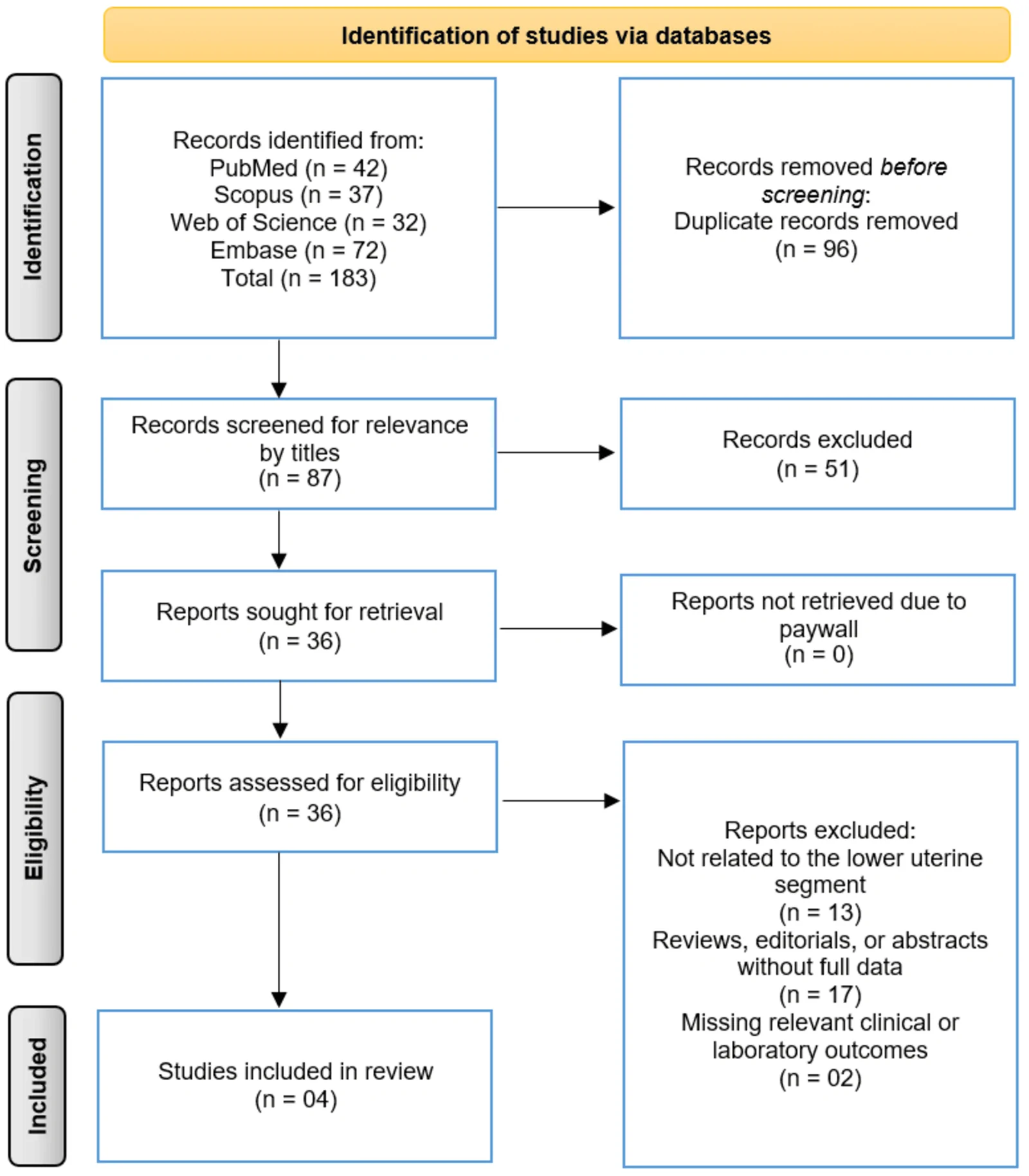
PRISMA 2020 flow diagram of study selection PRISMA: Preferred Reporting Items for Systematic Reviews and Meta-Analyses

Study Characteristics

The four included studies were retrospective comparative cohorts conducted in China [8,10,11] and Japan [9], with 91-346 women each (Table 2). Three enrolled women with pernicious previa or previa with accreta spectrum [8,10,11], and one enrolled women with previa in general [9].

**Table 2:** Characteristics of included studies PAS, placenta accreta spectrum; PLBCS, parallel loop binding compression suture; VCS, vertical compression sutures; TPCS, transverse parallel compression suture; ET, endotracheal; NS, not statistically significant; ROBINS-I, Risk of Bias in Non-randomized Studies - of Interventions

Study	Country	Design	Population (n)	Intervention versus comparator (n)	Key findings	ROBINS-I overall
Fu et al., 2021 [8]	China	Retrospective cohort	Pernicious previa with increta/percreta (124)	PLBCS (38) versus figure-of-eight sutures (86)	Blood loss of 2152.6 versus 2960.5 mL (P = 0.02); 3/38 hysterectomies with PLBCS	Low
Kawakami et al., 2024 [9]	Japan	Retrospective cohort	Placenta previa (117)	VCS + balloon (29) versus balloon (41) versus conventional (47)	Fewer additional procedures; lower postoperative loss than balloon; higher total loss than conventional (more PAS)	Serious (confounding)
Huang et al., 2025 [10]	China	Retrospective comparative	Pernicious previa with severe PAS (91)	Double-row transfixion (47) versus aortic balloon + sutures (44)	Hysterectomy 0/47 versus 2/44 (NS); less blood loss and shorter surgery	Serious (confounding)
Lin et al., 2026 [11]	China	Retrospective, propensity-matched	Complete previa (346; 106 matched)	TPCS + ET tube (53) versus uterine packing (53)	Blood loss of 792.5 vs 1220.8 mL (P = 0.029); shorter surgery and stay	Low

Hemostasis and Blood Loss

Two studies reported mean blood loss, and both found lower loss with the suture than with their own comparator (Figure 2) [8,11]: the mean difference was -808 mL (95% CI: -1371 to -245) against figure-of-eight sutures [8] and -428 mL (95% CI: -812 to -44) against uterine packing [11]. Huang et al. likewise reported less blood loss with double-row transfixion than with aortic balloon plus conventional sutures [10]. In Kawakami et al., total blood loss was higher in the Hot-Dog group (vertical compression sutures with balloon) than in the conventional group, but the Hot-Dog group included more women with PAS, in whom the adherent placenta bleeds more heavily, and the groups also differed in placental location; this baseline imbalance, rather than the technique, is the likely explanation [9]. The Hot-Dog group required no backup procedures and had lower postoperative blood loss than the balloon group [9]. Operative time was shorter with the suture than with the respective comparator in three studies [8,10,11]. Because each study used a different technique and comparator, these results are separate within-study comparisons and were not combined.

**Figure 2:**
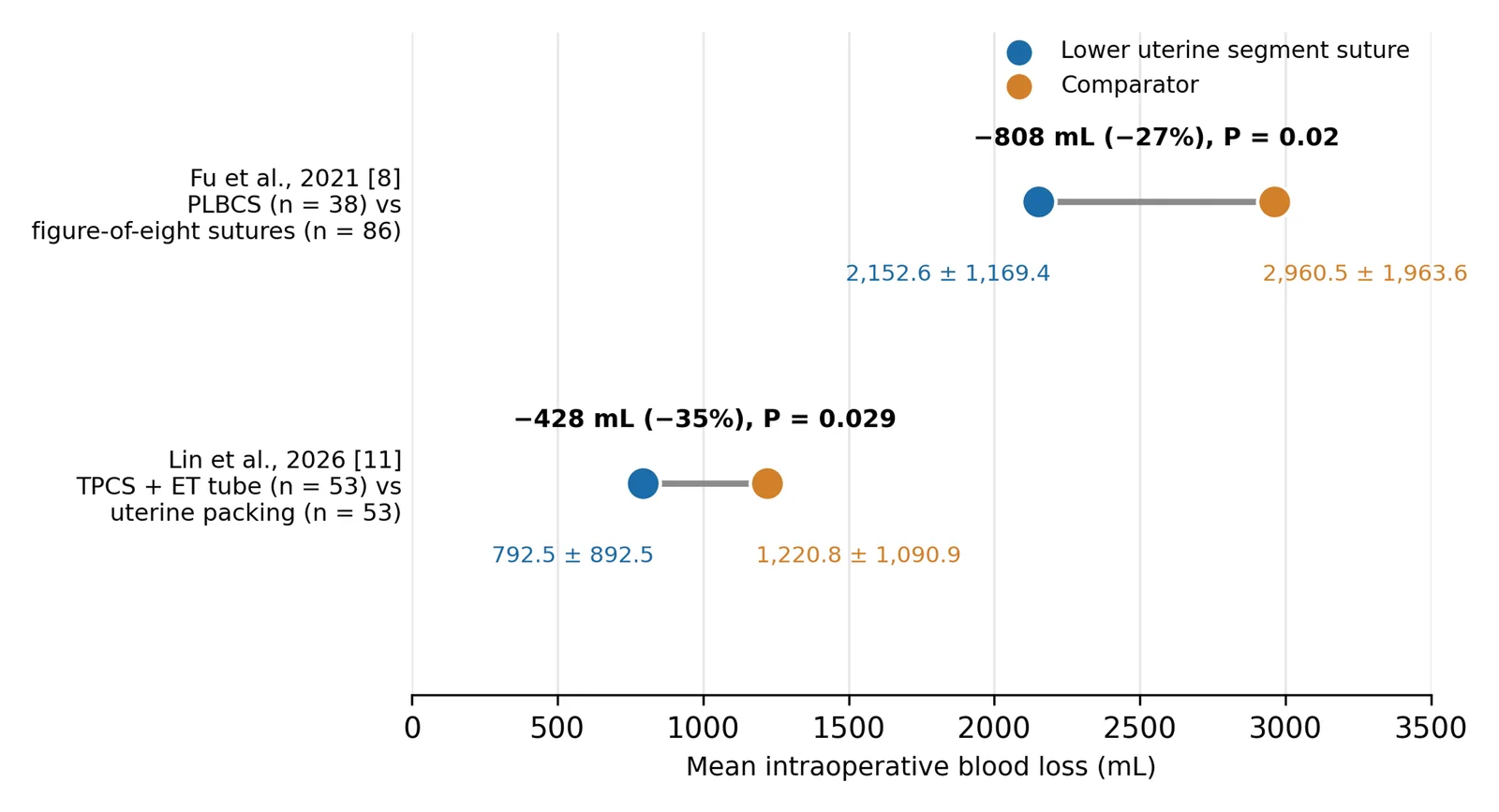
Mean intraoperative blood loss (±SD) in the suture group versus the comparator group Percentage differences were calculated from reported means; P values are from the original studies [8,11]. Mean differences (95% CI, Welch method) calculated from reported means, SDs, and group sizes: Fu et al., -808 mL (-1371 to -245) [8]; Lin et al., -428 mL (-812 to -44) [11] PLBCS, parallel loop binding compression suture; TPCS, transverse parallel compression suture; ET, endotracheal; CI, confidence interval; SD, standard deviation

Uterine Preservation

Hysterectomy was reported as a separate outcome in two studies. Huang et al. reported no hysterectomies among 47 women treated with the suture and two among 44 (4.5%) in the comparator group, a difference that was not statistically significant [10], and Fu et al. reported three hysterectomies among 38 suture-treated women (7.9%) [8]. Kawakami et al. [9] and Lin et al. [11] did not report hysterectomy separately but reported fewer additional or repeat procedures with the sutures. Hysterectomy was therefore infrequent among suture-treated women in the studies that reported it.

Risk of Bias

The ROBINS-I assessment is shown in Figure 3. Fu et al. [8] and Lin et al. [11] were at low risk of bias in all domains. Kawakami et al. [9] and Huang et al. [10] were at serious overall risk, driven by confounding. In both, the selection of participants was rated moderate. Kawakami et al. [9] were also rated moderate for outcome measurement and the selection of the reported result and Huang et al. [10] for missing data and outcome measurement. Domain-level judgements for each study are shown in Figure 3.

**Figure 3:**
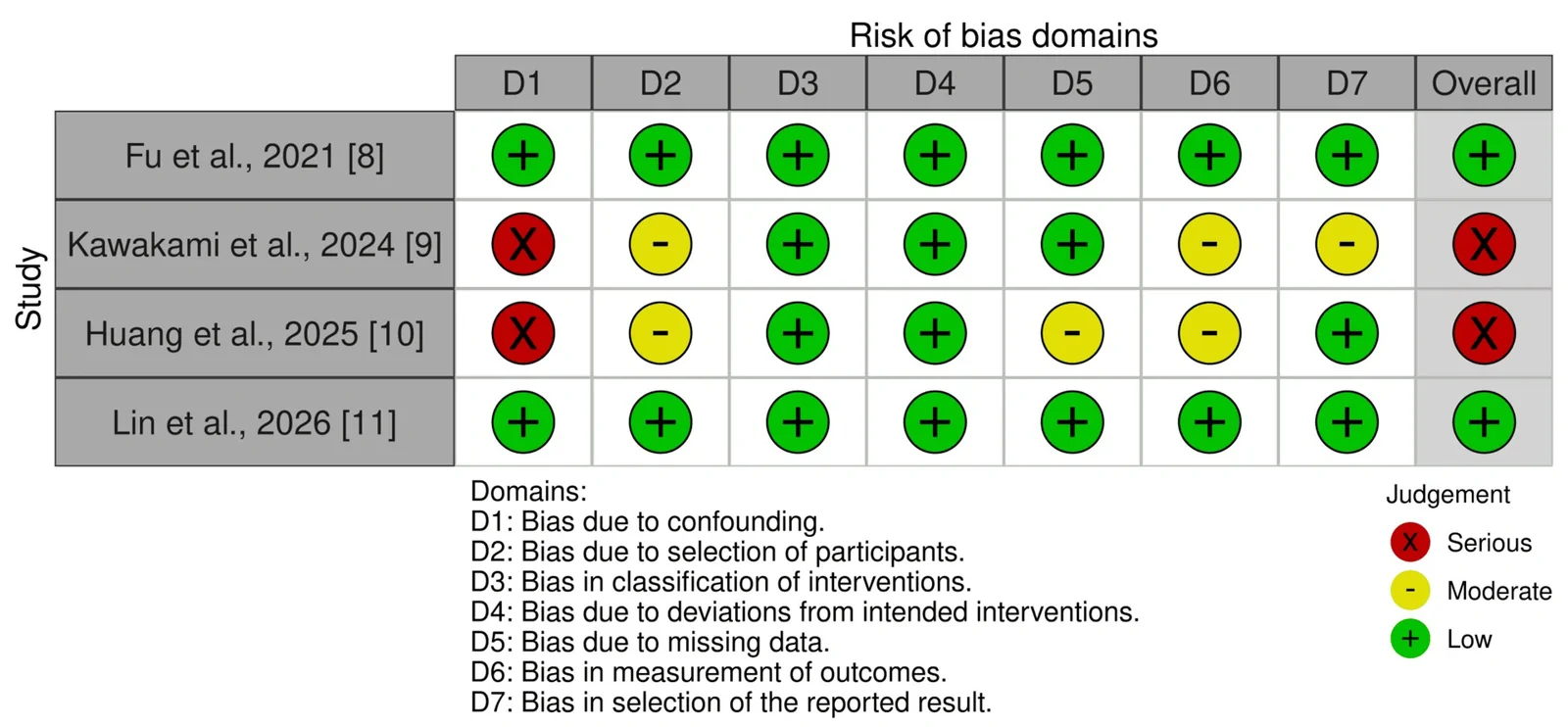
Risk of bias of the included studies assessed with ROBINS-I ROBINS-I: Risk of Bias in Non-randomized Studies - of Interventions

Discussion

In this review of comparative studies, lower uterine segment-directed sutures were associated with lower blood loss, shorter operations, and fewer additional procedures than the alternatives. Hysterectomy was infrequent in the two studies that reported it. These findings come from four retrospective cohorts, two at low and two at serious risk of bias, so they should be regarded as hypothesis-generating rather than confirmatory.

Findings of the Included Studies

The direction of effect is consistent. Fu et al. found lower blood loss with a parallel loop binding suture than with figure-of-eight sutures in women with previa and increta [8]. Lin et al. found shorter operations, lower blood loss, and shorter hospitalization after matching on baseline characteristics [11]. Huang et al. found no hysterectomy with double-row transfixion, against two hysterectomies with aortic balloon plus conventional sutures [10]. The latter difference was not statistically significant, and the study was small. The three studies used different comparators, which limits direct comparison, yet each favored the suture on at least one measure of blood loss or operative burden.

Context From Earlier and Non-included Studies

The findings fit earlier work. Hayman et al. [3] and the overview by Matsubara et al. [4] established compression sutures as a uterus-sparing option, largely for atony. Conventional techniques may not suit the thin lower segment, which motivated lower-segment-specific designs [5]. Zhao et al. reported hemostasis in 30 of 32 women with a transverse parallel suture and no hysterectomy, against two hysterectomies in the conventional group [5]. This is the same direction of effect as in our included studies, although that study falls outside the review window.

The following single-arm series were excluded from the review because they had no comparison group; they are cited only for context and are not part of the evidence base. Zhu et al. described a modified suture with balloon in 10 patients [12]. Xia et al. reported hemostasis in all 48 women treated with a combined suture, with no hysterectomy, although fewer than two-thirds of these women had previa [13]. Yin et al. combined a spiral suture with aortic balloon occlusion in morbidly adherent previa, which makes the contribution of the suture hard to isolate [14]. Shimizu et al. applied a modified Nausicaa suture to 45 women; the suture alone sufficed in 34, and 11 needed additional hemostatic procedures [15]. Taken together, these reports suggest high but not universal success, and they underline that adjunctive measures are often needed.

The role of tamponade deserves attention. Balloon tamponade has been used in previa [16], and Kawakami et al. suggest that vertical sutures may hold the balloon in place [9]. Whether suture plus balloon outperforms either alone cannot be resolved here, because the groups differed in PAS prevalence and placental location.

Uterine compression sutures themselves are well established: multiple square sutures at cesarean delivery [17] and combined longitudinal and transverse compressive sutures for atony [18] were shown to control bleeding and avoid hysterectomy more than two decades ago. The contribution of the present review is comparative evidence for variants directed specifically at the lower segment in placenta previa.

Clinically, the findings support considering a lower-segment suture early in cesarean for previa, especially where angiographic resources are limited. Huang et al. pointed out that avoiding aortic balloon placement lowers cost and complexity [10]. Sutures are inexpensive and need no special equipment, so they may be adopted more readily in resource-limited settings where interventional radiology is unavailable. They require surgeon familiarity with the technique and with bladder dissection, and tight sutures may carry risks such as ischemia or, in thin segments, tearing.

Knowledge gaps remain. No randomized trial has compared suture techniques in previa, and the comparators differ widely across studies. Outcomes are reported inconsistently, particularly hysterectomy and long-term fertility. Few studies follow women for menstrual or reproductive outcomes, and none has enough power to detect differences in rare events such as hysterectomy or maternal death. Reports should also describe the depth, number, and placement of sutures, because technique descriptions vary and are needed for reproducibility. Future work should use standardized definitions of hemostatic success, stratify by PAS status, and include fertility follow-up.

Limitations

This review has several limitations. Only four studies were included, all retrospective, and two were at serious risk of bias from confounding by PAS status or treatment era. Techniques, comparators, and outcome definitions differed, which precluded meta-analysis and limited direct comparison. Hysterectomy and fertility outcomes were not reported uniformly. Restricting inclusion to comparative designs from the last five years excluded informative single-arm series and an earlier comparative study. Four databases were searched, and the certainty of evidence was not formally graded. Screening, data extraction, and risk-of-bias assessment were performed by a single reviewer, which may have introduced errors.

## Conclusions

Uterine compression sutures are an established uterus-sparing technique, and variants directed at the lower uterine segment appear to be a useful, low-cost hemostatic option during cesarean delivery for placenta previa. They were associated with less blood loss, shorter operations, and few hysterectomies where reported. The evidence is limited to four retrospective comparative cohorts, so prospective studies with standardized outcome definitions and fertility follow-up are needed.

## Appendices

Table 3 shows the complete database-specific strategies. 

**Table 3:** Database-specific search strategies (last search 12 September 2026)

Database	Search strategy
PubMed	("lower uterine segment"[tiab] OR "LUS"[tiab] OR "isthmic"[tiab] OR "lower segment"[tiab]) AND (suture*[tiab] OR suturing[tiab] OR "compression technique*"[tiab] OR "hemostatic technique*"[tiab]) AND ("placenta previa"[MeSH Terms] OR "placenta praevia"[tiab] OR "placenta accreta"[tiab] OR "placenta increta"[tiab] OR "placenta percreta"[tiab] OR "placenta accreta spectrum"[tiab] OR "PAS"[tiab] OR "morbidly adherent placenta"[tiab]) AND ("cesarean"[tiab] OR "caesarean"[tiab] OR "cesarean section"[MeSH Terms] OR "cesarean delivery"[tiab]) AND ("hemostasis"[tiab] OR "haemostasis"[tiab] OR "hemostatic success"[tiab] OR "bleeding control"[tiab] OR "blood loss"[tiab] OR "hysterectomy"[tiab] OR "uterine preservation"[tiab] OR "uterine-sparing"[tiab] OR "fertility preservation"[tiab])
Scopus	TITLE-ABS-KEY(("placenta previa" OR "placenta praevia" OR "placenta accreta") AND ("lower uterine segment" OR "lower segment") AND (suture* OR transfixion) AND (cesarean OR caesarean)) AND PUBYEAR > 2020
Web of Science	TS=(("placenta previa" OR "placenta praevia" OR "placenta accreta") AND ("lower uterine segment" OR "lower segment") AND (suture* OR transfixion) AND (cesarean OR caesarean)); publication years 2021-2026
Embase	("placenta previa"/exp OR "placenta previa":ti,ab OR "placenta praevia":ti,ab OR "placenta accreta":ti,ab) AND ("lower uterine segment":ti,ab OR "lower segment":ti,ab) AND ("suturing method"/exp OR suture*:ti,ab OR transfixion:ti,ab) AND ("cesarean section"/exp OR cesarean:ti,ab OR caesarean:ti,ab) AND (2021-2026)/py
